# Sex-specific modulation of gut microbiota by empagliflozin contributes to renoprotection in diabetic kidney disease mice

**DOI:** 10.3389/fmicb.2026.1790953

**Published:** 2026-06-05

**Authors:** Jundong He, Jiaxin Liu, Heming Zhang, Yiting Wang

**Affiliations:** 1 Department of Endocrinology and Metabolism, The First People’s Hospital of Yunnan Province, The Affiliated Hospital of Kunming University of Science and Technology, Kunming, Yunnan, China; 2 School of Medicine, Kunming University of Science and Technology, Kunming, Yunnan, China; 3 Department of Gastroenterology, The First People’s Hospital of Yunnan Province, The Affiliated Hospital of Kunming University of Science and Technology, Kunming, Yunnan, China

**Keywords:** diabetic kidney disease, empagliflozin, gut microbiota, renoprotection, sex differences

## Abstract

**Objectives:**

This study aimed to explore the sex-specific regulatory effects of empagliflozin on gut microbiota in male and female diabetic kidney disease (DKD) mice, and to elucidate the underlying renoprotective mechanisms.

**Methods:**

Four-week-old db/db mice and C57 mice were randomly assigned to six groups. Following 8 weeks of empagliflozin gavage, serum metabolic indices and urinary albumin-to-creatinine ratio (ACR) were measured. Renal pathological alterations were evaluated via hematoxylin-eosin and Masson’s trichrome staining. Gut microbiota diversity and community composition were analyzed using 16S rRNA gene sequencing, and Spearman’s rank correlation analysis was performed to assess associations between dominant microbial taxa and metabolic/renal parameters.

**Results:**

ACR levels were significantly elevated in db/db mice compared to sex-matched C57 controls, with male db/db mice exhibiting significantly higher ACR than females. Empagliflozin significantly reduced ACR in db/db mice, albeit ACR remained significantly higher in males than females post-treatment. Meanwhile, empagliflozin ameliorated glomerular hypertrophy and mesangial proliferation. Pronounced sexual differences were observed in gut microbiota diversity of db/db mice, with female mice displaying significantly higher microbial richness than males. Empagliflozin effectively reshaped gut microbiota composition and alleviated microbial dysbiosis in db/db mice, with these regulatory effects showing distinct sex specificity. Furthermore, several dominant microbial taxa (e.g., *Rikenellaceae_RC9_gut_group*, *Parabacteroides*, *Klebsiella*) were identified to be significantly correlated with ACR, and these correlations were sex-dependent.

**Conclusion:**

Empagliflozin significantly reduces ACR levels and modulates gut microbial richness and diversity in db/db mice, with distinct sex-specific effects on microbiota composition. Ultimately, these findings suggest that empagliflozin may exert renoprotective effects in DKD by reshaping gut microbial community structure.

## Introduction

Diabetic kidney disease (DKD) is a leading microvascular complication of diabetes mellitus and the primary cause of end-stage kidney disease (ESKD) in developed countries, imposing a heavy economic burden on public health systems (Martinez Leon et al., 2026). As a rapidly progressive condition, our prior study demonstrated that biopsy-proven DKD patients experience an annual glomerular filtration rate (GFR) decline of approximately 8.1 mL/(min⋅1.73 m^2^) (Wang et al., 2019). DKD pathogenesis is complex, involving interconnected pathways such as metabolic dysregulation, hemodynamic disturbances, inflammatory responses, and oxidative stress (Jung and Yoo, 2022). Notably, a growing body of research has highlighted sex as a critical modifier of DKD progression and treatment outcomes. Studies in type 2 diabetes mellitus (T2DM) patients have increasingly suggested that females may be at elevated risk of DKD progression (Maric-Bilkan, 2020), and a Japanese study reported a higher annual estimated GFR (eGFR) decline in females (3.5%) than in males (2.0%) (Kajiwara et al., 2016). Our preliminary work further showed no significant sex differences in baseline renal pathological lesions or long-term ESKD risk among biopsy-confirmed DKD patients (Wang Y. et al., 2021). Crucially, none of the patients in these prior studies received sodium-glucose cotransporter 2 (SGLT2) inhibitors, and to date, no research has explored the sex-specific effects of this drug class in DKD.

The gut microbiota plays a pivotal role in the pathogenesis and progression of DKD. Clinical studies have documented that DKD patients exhibit marked reductions in gut microbial richness and diversity, an elevated *Firmicutes/Bacteroidetes* ratio, depleted abundances of short-chain fatty acid (SCFA)-producing taxa (e.g., *Faecalibacterium*), and increased levels of opportunistic pathogens (e.g., *Escherichia coli* and *Enterococcus*) (Zali et al., 2025). In preclinical models, fecal microbiota transplantation (FMT) from healthy donors to DKD mice restored gut microbial diversity, reduced harmful bacterial abundances, improved glycemic control, lowered the urine protein-to-creatinine ratio, and significantly attenuated pathological lesions including glomerulosclerosis and renal fibrosis (Shang et al., 2022). Mechanistically, gut microbial dysbiosis in DKD can trigger chronic inflammation and activate the intrarenal renin-angiotensin system (RAS), thereby inducing renal injury (Lu et al., 2020). Furthermore, the gut microbiota modulates host metabolism, and its dysregulation generates aberrant metabolites that accelerate DKD progression. For instance, trimethylamine (TMA)/trimethylamine N-oxide (TMAO) and bile acids have been linked to adverse clinical outcomes (Xu et al., 2024; Yang et al., 2022). Lastly, gut microbial dysbiosis may compromise intestinal barrier function, facilitating the translocation of bacteria and their metabolites into the systemic circulation, which activates immune responses and exacerbates renal inflammation and injury (Linh et al., 2022).

SGLT2 inhibitors, as novel antidiabetic agents, confer pleiotropic benefits beyond glycemic control, including robust cardiovascular and renoprotective effects. Clinical trials have validated that SGLT2 inhibitors significantly reduce proteinuria, decelerate GFR decline, and even mitigate the risk of ESKD in DKD patients (Kashihara et al., 2020), marking a landmark advancement in DKD therapeutics. Notably, the renoprotective effects of SGLT2 inhibitors mediated by dynamic modulation of gut microbial composition have emerged as a central research focus in DKD over recent years (Shi et al., 2023). For instance, empagliflozin has been shown to ameliorate T2DM-associated nephropathy by reshaping the gut microbiota, specifically by reducing lipopolysaccharide producing taxa and enriching SCFA-producing bacteria (Deng et al., 2022). Wu et al. (2023) further reported that the renoprotective effect of dapagliflozin in DKD may correlate with time-dependent improvements in gut microbiota, a process potentially mediated by dapagliflozin-induced modifications to the bile acid pool and the drug’s inherent antioxidant properties. Despite these findings, the interplay between the gut microbiota and SGLT2 inhibitors remains controversial. For example, one study demonstrated that treatment with a potent dual SGLT1/2 inhibitor improved glycemic control in rodent models without eliciting significant alterations in gut microbial diversity (Du et al., 2018).

A recent multi-omics study by Billing et al. (2024) demonstrated that SGLT2 inhibitors remodel gut microbial metabolism, reduce the production of microbiota-derived uremic toxins, and establish a beneficial metabolic communication network between the gut, kidney, and cardiovascular system. However, most previous studies have focused on the overall effects of SGLT2 inhibitors and neglected potential sex-specific differences in therapeutic responses, especially in the early-stage of DKD with mild renal injury. In the present study, we aimed to elucidate the effects of the SGLT2 inhibitor empagliflozin on gut microbiota composition and diversity in DKD mice, as well as whether such effects are sex-dependent. Enhanced understanding of the interplay between SGLT2 inhibitors and the gut microbiota is critical for optimizing therapeutic strategies for diabetes mellitus and its associated complications.

## Materials and methods

### Animals and grouping

Four-week-old db/db mice and C57 mice were purchased from Beijing HFK Bioscience Co., Ltd. (Beijing, China). Following a 1-week acclimatization period, the mice were randomly assigned to six groups: female C57 group (NC.F, *n* = 8), male C57 group (NC.M, *n* = 9), female db/db group (DM.F, *n* = 6), male db/db group (DM.M, *n* = 8), female db/db + empagliflozin group (Empa.F, *n* = 8), and male db/db + empagliflozin group (Empa.M, *n* = 7). All mice were housed under standardized conditions with a constant temperature and humidity, and had *ad libitum* access to standard chow and water. Once db/db mice developed manifestations of polydipsia and polyuria, random blood glucose levels were monitored. The diabetic model was considered successfully established when three consecutive random blood glucose readings exceeded 16.7 mmol/L.

Subsequent to successful diabetic model establishment, mice in the Empa.F and Empa.M groups were administered empagliflozin by gavage at a dose of 10 mg/kg/day for 8 consecutive weeks. Urine and peripheral blood samples were collected 24 h prior to euthanasia, and kidney tissues were harvested immediately post-euthanasia. The mice were sacrificed by cervical dislocation. This experimental protocol was approved by the Animal Ethics Committee of Kunming University of Science and Technology (Approval Number: AP-KUST-202412020011), and all procedures strictly complied with the Guide for the Care and Use of Laboratory Animals.

### Measurement of biochemical parameters

Serum levels of total cholesterol (Chol), triglycerides (TG), high-density lipoprotein (HDL), low-density lipoprotein (LDL), glycated serum protein (GSP), urea nitrogen (Urea), and creatinine (Cr) were measured with a TBA-FX8 automatic biochemical analyzer. Additionally, the urinary albumin-to-creatinine ratio (ACR) was determined using the same analyzer. All assays were performed in strict accordance with the manufacturer’s protocol.

### Masson’s trichrome and hematoxylin-eosin (HE) staining

Fresh kidney tissues were dehydrated, embedded in paraffin, and sectioned prior to Masson’s trichrome and hematoxylin-eosin (HE) staining. Staining was conducted using a Masson’s trichrome staining kit (G1340, Solarbio Life Sciences, Beijing, China) and a HE staining kit (G1120, Solarbio Life Sciences, Beijing, China), strictly in accordance with the manufacturer’s instructions.

### Collection of mouse fecal samples

Fecal samples were collected from each mouse 48 h prior to sacrifice. Mice were individually housed in cages lined with sterile filter paper, and fecal pellets were collected via the tail-lifting reflex. In cases where mice failed to defecate spontaneously, gentle abdominal massage with a sterile cotton swab was applied to induce defecation. Fecal pellets (5–6 per mouse) were collected immediately upon defecation and stored at −80°C in cryopreservation tubes until subsequent analysis.

### PCR amplification and 16S rRNA sequencing

Fecal DNA was extracted with the QIAGEN QIAamp PowerFecal Pro DNA Kit (Cat. No. 51804, QIAGEN, Hilden, Germany) strictly in accordance with the manufacturer’s instructions. After purification, the DNA samples were sent to Novogene Co., Ltd. (Beijing, China) for PCR amplification of the 16S rRNA gene V3-V4 hypervariable regions and subsequent high-throughput sequencing.

### Sequencing data analysis

Raw sequencing data were filtered and trimmed to generate high-quality clean reads. Operational Taxonomic Units (OTUs) were clustered, and taxonomic annotation was conducted based on the clean reads. Alpha diversity (intra-sample microbial diversity) was assessed using the Chao1 (richness) and Shannon (richness and evenness) indices. Beta diversity (inter-sample microbial community differences) was visualized via Principal Coordinate Analysis (PCoA) using Bray-Curtis distance and unweighted UniFrac metrics. Linear Discriminant Analysis Effect Size (LEfSe) was employed to identify taxonomic differences in microbial communities between groups. Additionally, Spearman’s rank correlation analysis was performed to evaluate associations between dominant microbial taxa and biochemical/renal parameters. Microbial functional potential was predicted using the Tax4Fun tool, focusing on KEGG Level 2 pathways. All gut microbiota bioinformatics analyses were conducted on the NovoMagic cloud platform,^1^ developed by Novogene. Statistical tests including Student’s *t*-test, SIMPER, MetaStat, LEfSe, ANOSIM, and MRPP were utilized to determine significant differences in microbial species composition and community structure across groups. A two-tailed *P*-value < 0.05 was considered statistically significant, and the False Discovery Rate (FDR) was used to correct for multiple testing.

### Statistical analysis

Quantitative data were expressed as mean ± standard deviation (SD). For comparisons between two groups, the Mann-Whitney U test was used. For multiple group comparisons, one-way analysis of variance (ANOVA) was conducted, followed by Tukey’s *post-hoc* test for pairwise comparisons. Statistical significance was set at *P* < 0.05. All statistical analyses were performed using GraphPad Prism software (Version 10.0.0; San Diego, CA, United States).

## Results

### Effects of empagliflozin on metabolic and renal parameters in diabetic mice

**FIGURE 1:**
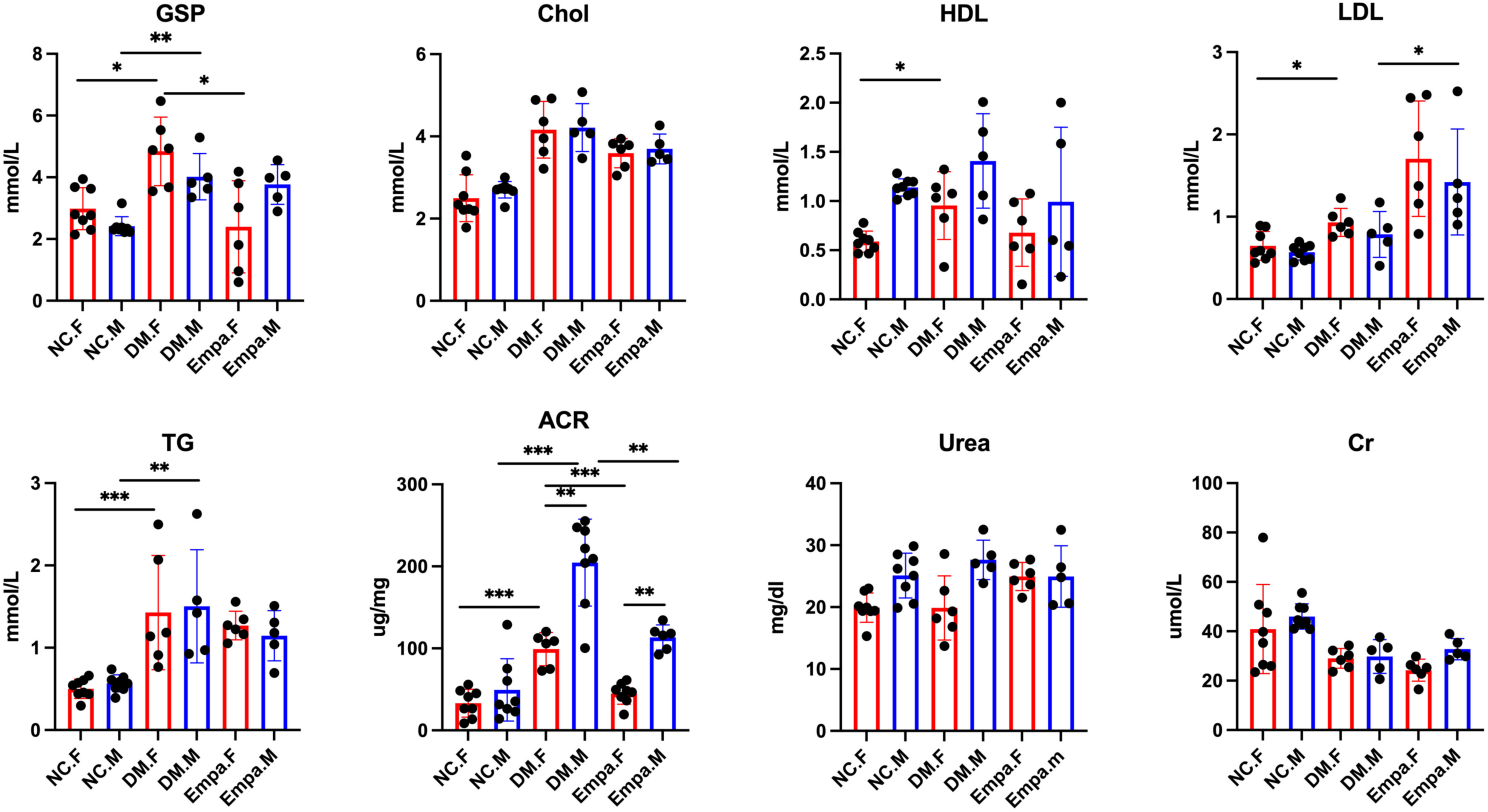
Serum and urinary parameter profiles of mice in each group. Abbreviations: GSP, glycated serum protein; Chol, cholesterol; HDL, high-density lipoprotein; LDL, low-density lipoprotein; TG, triglyceride; ACR, albumin-to-creatinine ratio; Urea, urea nitrogen; Cr, creatinine. Sample sizes for serum measurements: NC.F, *n* = 8; NC.M, *n* = 8; DM.F, *n* = 6; DM.M, *n* = 5; Empa.F, *n* = 6; Empa.M, *n* = 5. Sample sizes for urinary measurements: NC.F, *n* = 8; NC.M, *n* = 8; DM.F, *n* = 6; DM.M, *n* = 8; Empa.F, *n* = 8; Empa.M, *n* = 6. *P < 0.05, **P < 0.01, ***P < 0.001.

To evaluate the effects of empagliflozin on metabolic profiles and renal function-related parameters in db/db mice, we measured a panel of serum metabolic indices and urinary renal function markers (Supplementary Table 1). GSP levels were significantly elevated in both male and female db/db mice relative to sex-matched C57 control groups, confirming the successful establishment of the diabetic model. Empagliflozin treatment significantly lowered GSP levels in female db/db mice (Figure 1). Chol levels were increased in both male and female db/db mice compared to their C57 counterparts, and empagliflozin treatment induced a slight, non-statistically significant reduction in Chol levels (Figure 1). HDL levels were significantly higher in female db/db mice than in female C57 controls, with no significant alterations following empagliflozin intervention (Figure 1). LDL levels were significantly elevated in female db/db mice relative to female C57 controls; notably, empagliflozin treatment significantly increased LDL levels in male db/db mice (Figure 1). TG levels were significantly higher in both male and female db/db mice than in their respective sex-matched C57 controls (Figure 1).

ACR was significantly elevated in both male and female db/db mice compared to C57 controls, with male db/db mice exhibiting significantly higher ACR levels than females. Empagliflozin treatment significantly reduced ACR levels in both sexes, although ACR remained significantly higher in males than in females post-treatment (Figure 1). No significant differences in Cr or Urea levels were observed among all groups, indicating that the DKD model was in the early stage, with no overt changes in these parameters (Figure 1). Collectively, these results validate the successful establishment of the DKD model and demonstrate empagliflozin’s efficacy in reducing proteinuria.

### Effects of empagliflozin on renal tissue pathology in diabetic mice

**FIGURE 2:**
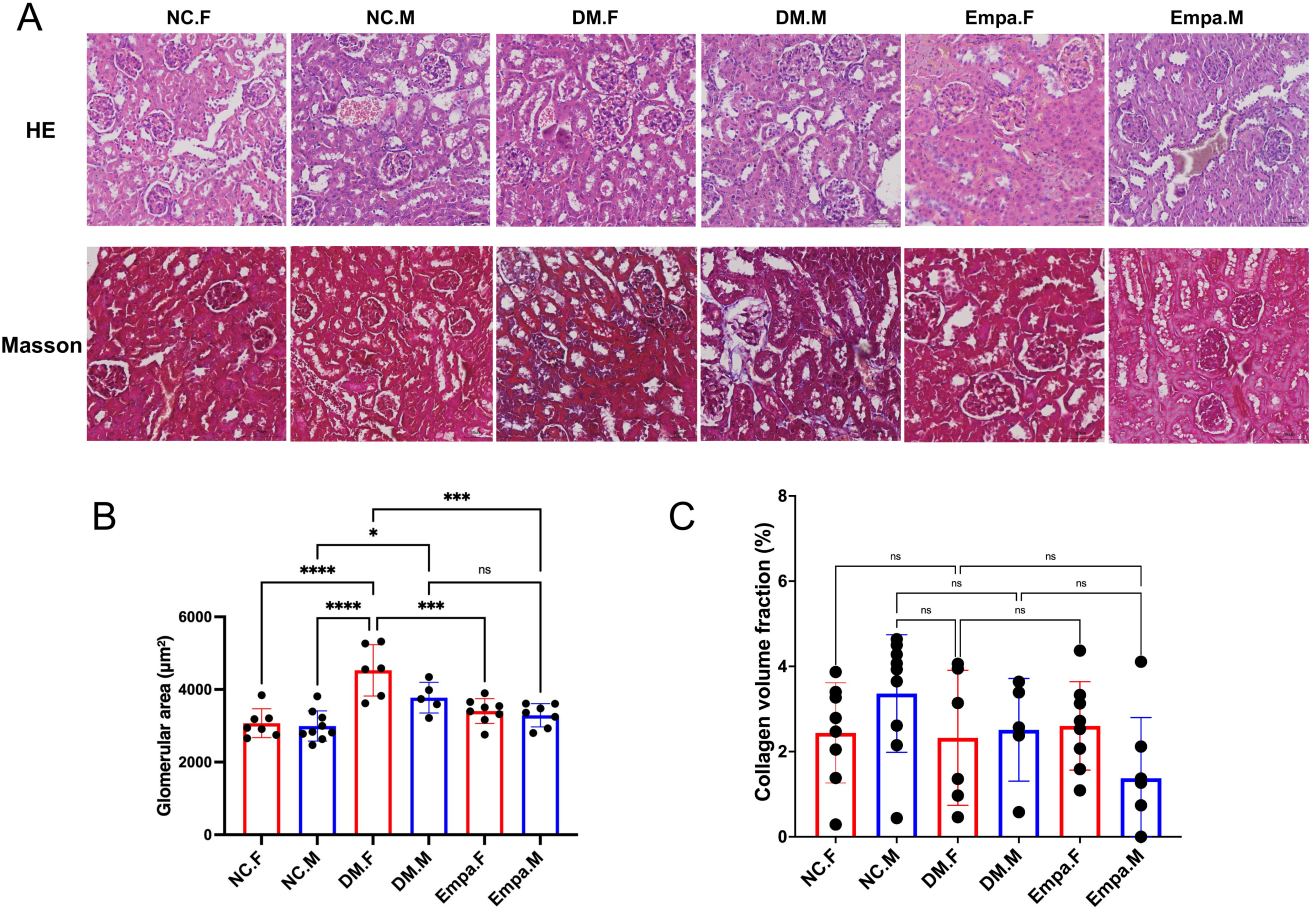
Effects of empagliflozin on renal morphology and collagen deposition in mice. **(A)** Representative HE staining (upper) and Masson’s trichrome staining (lower) images of kidney tissues (scale bar: 50 μm). **(B)** Quantitative analysis of glomerular cross-sectional area. **(C)** Quantitative analysis of collagen volume fraction. Data are presented as mean ± SD. Statistical analysis was performed using One-way ANOVA followed by Tukey’s multiple comparisons test. Sample sizes for HE staining: NC.F, *n* = 7; NC.M, *n* = 9; DM.F, *n* = 6; DM.M, *n* = 5; Empa.F, *n* = 8; Empa.M, *n* = 7. Sample sizes for Masson’s trichrome staining: NC.F, *n* = 8; NC.M, *n* = 9; DM.F, *n* = 6; DM.M, *n* = 5; Empa.F, *n* = 8; Empa.M, *n* = 7. *P < 0.05, ***P < 0.001, ****P < 0.0001, ns, not significant.

HE staining showed normal glomerular morphology in sex-matched C57 control mice, while both male and female db/db mice exhibited marked glomerular hypertrophy, mesangial proliferation, and glomerular basement membrane thickening, characteristic of early DKD. Quantitative analysis confirmed that the mean glomerular cross-sectional area was significantly increased in DM mice of both sexes compared with their respective sex-matched controls (*P* < 0.0001 for females, *P* < 0.05 for males). Empagliflozin treatment significantly reduced glomerular size in female DM mice (*P* < 0.001), but not in male DM mice. These results indicate that empagliflozin effectively ameliorates glomerular hypertrophy, a key early pathological feature of DKD (Figures 2A,B).

Masson’s trichrome staining showed no significant differences in collagen volume fraction among all groups. These results indicate no obvious tubulointerstitial fibrosis in this model, which is consistent with the unchanged urinary creatinine level and supports that the mice were at an early-stage of diabetic kidney injury without established renal fibrosis (Figures 2A,C). Previous studies have verified that inflammatory factors (TNF-α, IL-6) and fibrotic markers (TGF-β, collagen IV, fibronectin) are critical indicators for evaluating DKD progression (Hou et al., 2025). Although these molecular markers were not measured in the present study, our phenotypic and pathological data confirmed that this model accurately recapitulated early-stage DKD.

### Effects of empagliflozin on gut microbiota diversity in diabetic mice

**FIGURE 3:**
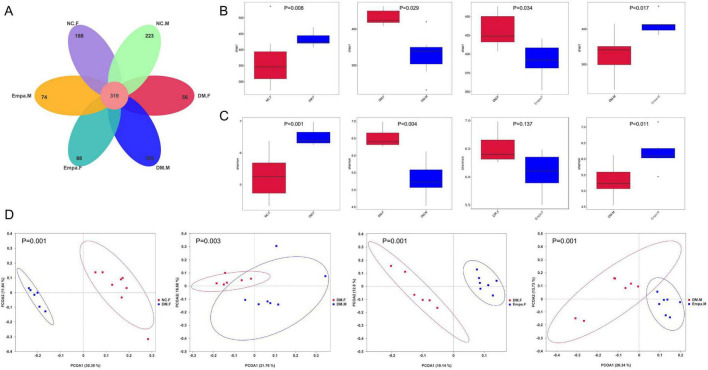
Gut microbiota diversity and richness profiles of mice in each group. **(A)** Venn diagrams illustrating OTU overlap and distribution across groups. **(B,C)** Alpha diversity assessed using the Chao1 (microbial richness) and Shannon (richness and evenness) indices. **(D)** Principal Coordinate Analysis (PCoA) based on the unweighted UniFrac distance metric. Sample sizes for gut microbiota profiling: NC.F, *n* = 8; NC.M, *n* = 8; DM.F, *n* = 6; DM.M, *n* = 7; Empa.F, *n* = 7; Empa.M, *n* = 7.

The number of OTUs increased progressively with sequencing depth and eventually plateaued, indicating sufficient sequencing data volume for subsequent analyses. Venn diagram analysis of OTU distribution across groups revealed that diabetes reduced OTU counts, while empagliflozin treatment further altered OTU abundance (Figure 3A).

Alpha diversity analysis was performed to assess gut microbial diversity. Chao1 index analysis demonstrated that gut microbial richness was significantly higher in female db/db mice than in both sex-matched C57 controls and male db/db mice. Following empagliflozin treatment, microbial richness decreased significantly in female db/db mice but increased markedly in male db/db mice (Figure 3B). Shannon index analysis showed that female db/db mice exhibited significantly higher microbial richness and evenness than controls and male db/db mice. Empagliflozin treatment induced a non-significant reduction in these parameters in females but a significant increase in males (Figure 3C). Collectively, these findings indicate pronounced sexual differences in gut microbial richness and evenness among db/db mice, with empagliflozin exerting opposing effects on the gut microbiota of male versus female db/db mice.

Beta diversity, which quantifies inter-community species diversity differences, was analyzed to characterize variations in microbial community structure across samples. PCoA was used to visualize these differences, and ANOSIM was performed to statistically validate group-level disparities in microbial community structure. Results showed significant differences in gut microbiota composition between female db/db mice and controls, as well as between female and male db/db mice. After empagliflozin treatment, both male and female db/db mice exhibited significant alterations in gut microbiota diversity compared to their untreated counterparts (Figure 3D).

Briefly, gut microbiota diversity in db/db mice displayed significant sexual differences. Empagliflozin robustly modulated the gut microbiota of db/db mice in a sex-dependent manner.

### Effects of empagliflozin on gut microbiota composition in diabetic mice

**FIGURE 4:**
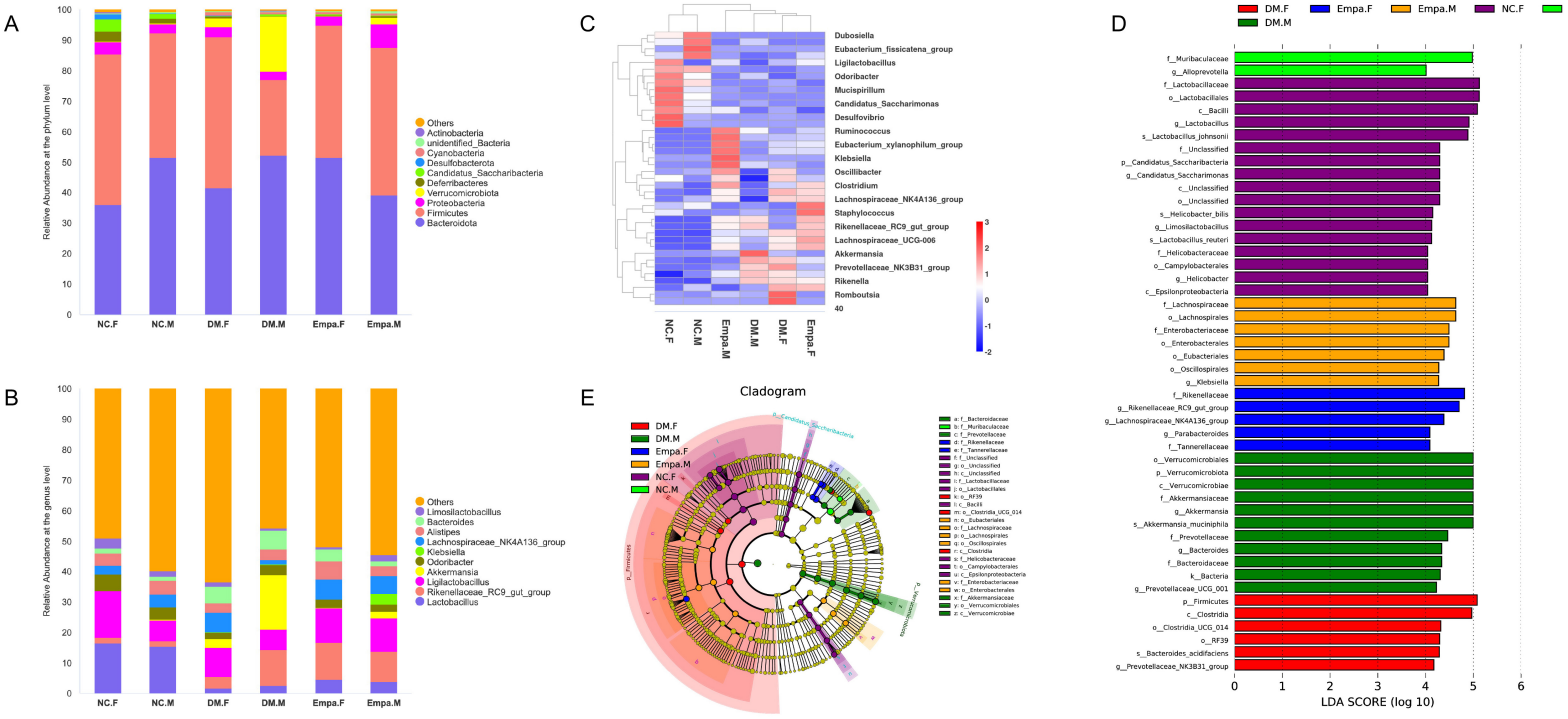
Gut microbiota community composition in each group. **(A,B)** Relative abundance of gut microbiota at the phylum and genus levels, respectively. **(C)** Heatmap of the top 20 significantly differential microbial genera across groups. **(D,E)** Linear discriminant analysis effect size (LEfSe) analysis: linear discriminant analysis (LDA) score plot **(D)** and cladogram depicting taxonomic cladistic relationships **(E)**. Gut microbiota data were obtained from the same mouse cohort as described in Figure 3.

To characterize the modulatory effects of empagliflozin on gut microbiota, we analyzed the microbial community composition. Bar graphs of dominant microbiota at the phylum level demonstrated that, relative to sex-matched C57 controls, both male and female db/db mice exhibited elevated abundance of *Verrucomicrobiota*, an effect reversed by empagliflozin treatment in both sexes. In male mice, *Firmicutes* abundance was reduced in db/db mice compared to C57 controls, and empagliflozin treatment restored *Firmicutes* levels. Notably, the drug also increased *Proteobacteria* abundance in male db/db mice (Figure 4A). At the genus level, bar graphs showed that *Akkermansia* abundance was elevated in both male and female db/db mice relative to C57 controls, while empagliflozin treatment decreased *Akkermansia* levels. In contrast, *Lactobacillus* abundance was diminished in db/db mice, and empagliflozin partially rescued *Lactobacillus* levels in both sexes (Figure 4B).

The top 20 significantly differential genera were selected for heatmap analysis, which revealed distinct clustering of dominant taxa among control groups, untreated db/db groups, and empagliflozin-treated groups, indicating marked shifts in dominant gut microbiota following empagliflozin intervention (Figure 4C). Notably, empagliflozin-treated female db/db mice were dominated by taxa such as *Rikenellaceae_RC9_gut_group* and *Lachnospiraceae_NK4A136_group*, whereas treated male db/db mice were enriched in *Klebsiella* and *Oscillibacter*, further confirming sex-specific effects of empagliflozin on gut microbiota (Figure 4C). LEfSe was performed to identify group-specific dominant taxa, with the LDA effect size threshold set at 4. Figures 4D,E illustrated the dominant taxa across the six groups, reflecting empagliflozin-induced alterations in core microbial communities. The female C57 group was dominated by *Lactobacillaceae*, *Lactobacillales*, *Bacilli*, *Lactobacillus*, and *Lactobacillus_johnsonii*, the male C57 group by *Muribaculaceae* and *Alloprevotella*. The female db/db group featured dominant taxa including *Firmicutes*, Clostridia, *Clostridia_UCG_014*, *RF39*, and *Bacteroides_acidifaciens*, while the male db/db group was characterized by *Verrucomicrobiales*, *Verrucomicrobiota*, *Verrucomicrobiae*, *Akkermansiaceae*, and *Akkermansia*. Following empagliflozin treatment, the female db/db group was enriched in *Rikenellaceae*, *Rikenellaceae_RC9_gut_group*, *Lachnospiraceae_NK4A136_group*, and *Parabacteroides*, whereas the treated male db/db group featured dominant taxa such as *Lachnospiraceae*, *Lachnospirales*, *Enterobacteriaceae*, *Enterobacterales*, and *Eubacteriales*.

In brief, these results indicate that empagliflozin markedly reshapes the gut microbiota of db/db mice, exerting distinct sex-specific modulatory effects.

### Correlations between gut microbiota, metabolic parameters, and functional potential

**FIGURE 5:**
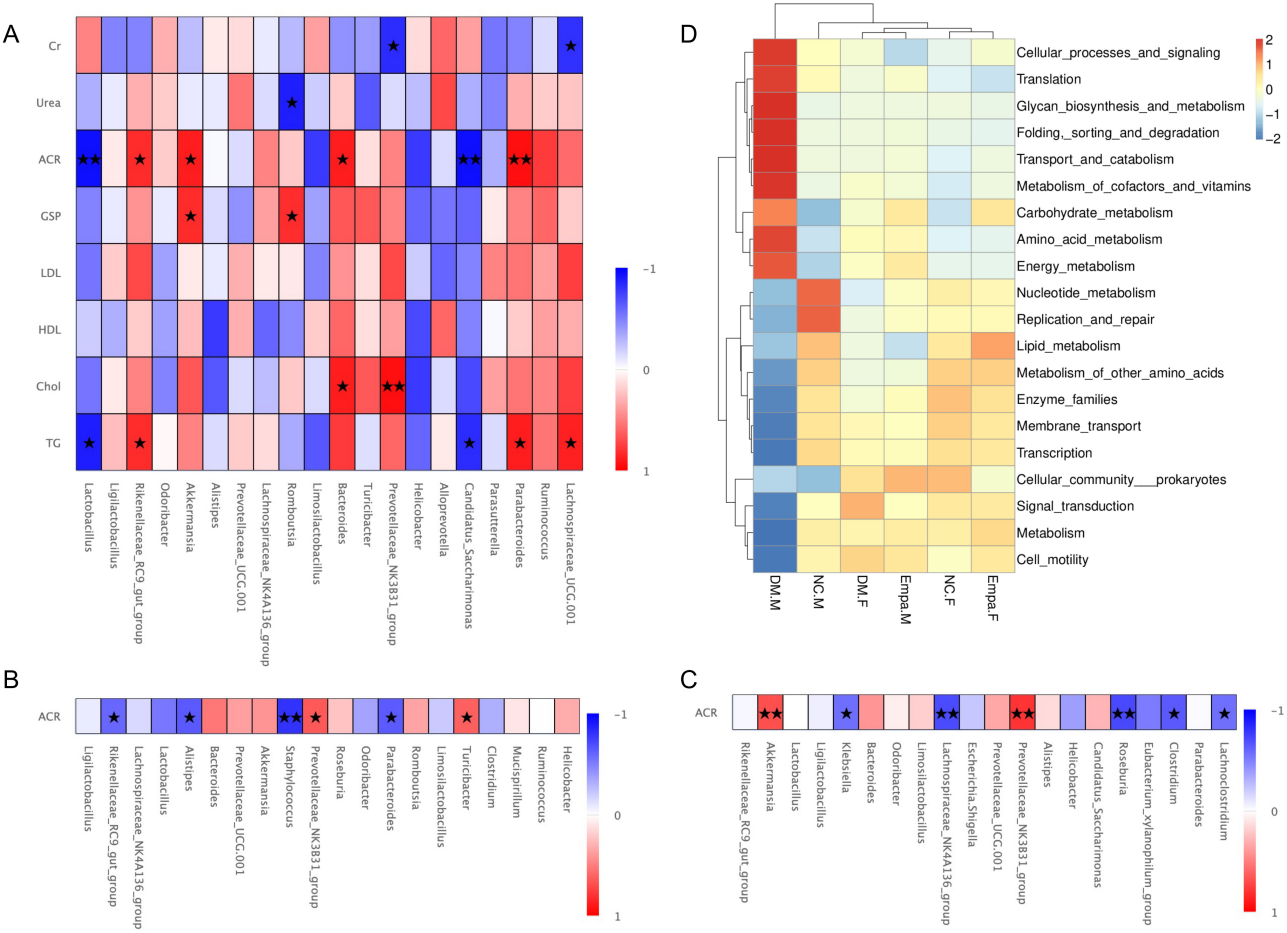
Correlations between gut microbiota and metabolic parameters, and microbial functional prediction. **(A–C)** Spearman’s rank correlation analysis between the top 20 significantly differential genera and serum metabolic/urinary renal parameters in all six groups **(A)**, female db/db mice (DM.F vs. Empa.F, **B**), and male db/db mice (DM.M vs. Empa.M, **C**). **(D**) Functional potential of gut microbiota predicted via the Tax4Fun tool, annotated against KEGG Level 2 pathways. Gut microbiota data were obtained from the same mouse cohort as described in Figure 3. *P < 0.05, **P < 0.01.

To identify key microbial taxa potentially mediating the renoprotective effects of empagliflozin, Spearman correlation analysis was conducted to explore associations between significantly altered gut microbial genera and relevant metabolic/renal parameters (Figure 5A). *Lactobacillus* exhibited a significant negative correlation with both ACR and TG levels. Notably, *Rikenellaceae_RC9_gut_group* was positively correlated with ACR and TG, while *Akkermansia* showed positive correlations with ACR and GSP. Furthermore, *Bacteroides* was positively associated with ACR and Chol, *Candidatus_Saccharimonas* displayed a negative correlation with ACR and TG, and *Parabacteroides* was positively correlated with ACR and TG. To further dissect the sex-specific correlations between empagliflozin-induced gut microbiota alterations and ACR levels, additional Spearman correlation analyses were performed in female (DM.F vs. Empa.F) and male (DM.M vs. Empa.M) db/db mice. In female db/db mice, *Prevotellaceae_NK3B31_group* and *Turicibacter* were significantly positively correlated with ACR, whereas *Rikenellaceae_RC9_gut_group*, *Alistipes*, *Staphylococcus*, and *Parabacteroides* exhibited a significant negative correlation with ACR (Figure 5B). In male db/db mice, *Akkermansia* and *Prevotellaceae_NK3B31_group* were significantly positively correlated with ACR, while *Klebsiella*, *Lachnospiraceae_NK4A136_group*, *Roseburia*, *Clostridium*, and *Lachnoclostridium* showed a significant negative correlation with ACR (Figure 5C).

Gut microbiota-derived metabolites exert multifaceted effects on the host. The functional potential of the gut microbiota was predicted using the Tax4Fun package, with results clustered based on functional differences (Figure 5D). Based on 16S rRNA sequencing data, we predicted the functional potential of gut microbiota using the Tax4Fun tool, and the results should be regarded as a scientific hypothesis rather than a direct experimental measurement, since no metabolomic or metagenomic experiments were performed to validate the predicted KEGG pathways. At the KEGG second-level pathway, male db/db mice exhibited significant upregulation of functional pathways including Cellular processes and signaling, Translation, and Glycan biosynthesis and metabolism, effects that were significantly reversed by empagliflozin treatment. In female db/db mice, empagliflozin treatment significantly increased the abundance of functional pathways such as Lipid metabolism and Metabolism of other amino acids compared to the untreated female db/db group.

## Discussion

DKD poses a severe threat to human health and survival, and the gut microbiota plays a pivotal role in its pathogenesis and progression. The present study investigated the effects of empagliflozin on the gut microbiota of DKD mice across sexes, revealing significant sexual differences in gut microbiota diversity between male and female db/db mice. Furthermore, empagliflozin exerted robust, sex-specific modulatory effects on the gut microbiota of these mice, effectively reshaping the microbial community to alleviate DKD-associated renal injury.

Gut microbiota diversity is a core indicator of intestinal microecological balance, with its perturbations playing a critical role in DKD pathogenesis. Numerous studies have documented that DKD patients display characteristic gut microbiota diversity changes closely linked to disease progression (Han et al., 2022; Shang et al., 2022). A systematic review and meta-analysis showed that gut microbial richness was markedly reduced in DKD patients relative to healthy individuals (Han et al., 2022). Notably, microbiota diversity exhibits dynamic shifts with DKD advancement, with higher diversity in advanced-stage than early-stage patients, a pattern attributed to the selective enrichment of harmful taxa and gradual depletion of beneficial ones during disease progression8. In our study, we observed that gut microbial richness was significantly higher in female db/db mice than in both control mice and male db/db mice. These findings reveal pronounced sexual differences in the gut microbiota of db/db mice, and the elevated microbial richness in female db/db mice may correlate with their lower urine ACR, potentially conferring a renoprotective effect.

Renal function and diabetic status collectively shape the gut microbial community structure (Koshida et al., 2023). For example, liraglutide treatment significantly increased the Simpson index and ameliorated renal pathological damage in DKD rats, implying that restored microbiota diversity may mediate its renoprotective effects (Yi et al., 2024). Likewise, pirfenidone reversed the decline in gut microbiota diversity in db/db diabetic mice and reduced the accumulation of diabetic ketoacidosis-related metabolic markers (e.g., 3-hydroxybutyric acid and acetoacetic acid) (Singh et al., 2020). In line with these findings, we observed that empagliflozin significantly reduced ACR levels and altered gut microbial richness and diversity in db/db mice, suggesting it may exert renoprotective effects by reshaping the gut microbial community structure. Collectively, alterations in gut microbiota diversity in DKD patients directly reflect intestinal microecological imbalance, contribute to renal injury onset and progression, and offer valuable insights for DKD assessment.

Specific alterations in gut microbiota composition constitute a core feature of microbial dysbiosis in DKD, with shifts in the abundance of distinct phyla and genera driving DKD progression via multiple mechanisms (Wang et al., 2025). Gut microbiota-derived SCFAs exert anti-inflammatory effects and delay DKD progression by alleviating glomerular hypertrophy and interstitial fibrosis, enhancing intestinal barrier integrity, and reducing the translocation of harmful substances into the bloodstream (Li et al., 2023). Furthermore, a Mendelian randomization study demonstrated that *Coprococcus 2* and *Defluviitaleaceae* conferred significant protective effects against DKD, whereas elevated abundances of *Bacteroidetes*, *Lachnoclostridium*, and *Veillonellaceae* markedly increased DKD risk (Yang et al., 2025). Zhang et al. (2023) characterized gut microbiota across DKD stages and found that genera including *Fusobacterium*, *Parabacteroides*, and *Ruminococcus gnavus* were significantly enriched in early DKD and accumulated progressively with disease advancement. Compared with the diabetes mellitus group, counts of *Agathobacter*, *Prevotella 9*, and *Roseburia* were markedly reduced in both early and advanced DKD (Li et al., 2023). Additionally, Wang et al. (2025) reported that advanced DKD patients had higher abundances of *Butyricimonas*, *Fusicatenibacter*, and *Barnesiella*, and lower *Allisonella* relative to early-stage patients, while DKD-susceptible individuals exhibited elevated *Fusobacterium* and reduced *Allisonella* and *Eubacterium*.

We observed significant enrichment of *Bacteroidetes* in male db/db mice; following empagliflozin treatment, *Lachnospirales* became dominant, a taxon potentially linked to SCFA synthesis and intestinal barrier maintenance. In female db/db mice, *Firmicutes* were markedly enriched, and empagliflozin treatment significantly increased the abundances of *Parabacteroides* and *Lachnospiraceae_NK4A136_group*, the latter of which may also participate in SCFA synthesis. Consistently, Zhang et al. (2023) reported aberrant abundances of *Parabacteroides* and *Firmicutes* in DKD, further noting that *Parabacteroides* correlated with DKD onset and progression, particularly in the early stage. Collectively, our findings revealed sexual differences in both the gut microbiota composition of DKD mice and the modulatory effects of empagliflozin on gut microbiota.

ACR is a key clinical indicator for assessing renal injury severity and disease progression in DKD. The gut microbiota regulates ACR levels via direct or indirect pathways, with underlying mechanisms confirmed by multiple clinical and basic studies (He et al., 2022; Lan et al., 2023). Clinical studies identified a significant correlation between gut microbiota composition and ACR in DKD patients: in early-stage DKD, the abundances of *Citrobacter farmeri* and *Syntrophaceticus schinkii* were positively correlated with ACR (He et al., 2022), while levels of *Agathobacter*, *Prevotella 9*, *Lachnospira*, and *Roseburia* correlated positively with eGFR and negatively with microalbuminuria, 24-h urinary protein excretion, and serum creatinine (Li et al., 2023). Notably, *Agathobacter* may serve as the most promising gut bacterial biomarker for distinguishing DKD stages (Zhang et al., 2023). Bidirectional Mendelian randomization analysis revealed that *Barnesiella*, *Butyricimonas*, *Desulfovibrio*, and *Hemophilus* exacerbated DKD, while *Slackia* and *Allisonella* prevented eGFR decline (Wang et al., 2025). Additionally, ten key bacterial genera were causally linked to DKD progression and susceptibility, encompassing harmful taxa (*Barnesiella*, *Butyricimonas*, *Desulfovibrio*, *Hemophilus*, *Bacteroides*, *Streptococcus*, *Ruminococcus 2*) and protective taxa (*Slackia*, *Allisonella*, *Akkermansia*) (Wang et al., 2025).

In the present study, *Prevotellaceae_NK3B31_group*, a dominant genus in female db/db mice, was positively correlated with ACR, whereas empagliflozin treatment reduced female db/db mouse ACR levels and enriched *Rikenellaceae_RC9_gut_group* and *Parabacteroides*, both of which were negatively correlated with ACR. In male db/db mice, the dominant genus *Akkermansia* was positively correlated with ACR; empagliflozin also reduced ACR in males and enriched *Klebsiella*, a genus negatively correlated with ACR. It should be emphasized that all the above associations are merely statistical covariations between gut microbial taxa and renal injury indicators, and no definitive causal relationship can be inferred from these correlation analyses alone. The potential regulatory effects of these microbial genera on renal injury and their direct causal link with empagliflozin mediated renoprotection still need to be verified by subsequent *in vivo* and *in vitro* intervention experiments (e.g., FMT, microbial knockout/overexpression). Notably, *Rikenellaceae_RC9_gut_group* and *Parabacteroides* serve as biomarkers for Sacubitril/Valsartan, a drug that improves renal function and modulates gut microbiota in DKD mice (Wang P. et al., 2022). Two systematic reviews and meta-analyses reported higher gut *Klebsiella* abundance in DKD patients than in healthy individuals (Hong et al., 2022; Wang Y. et al., 2021), with further reductions in mid-stage versus early-stage DKD, implying a link between *Klebsiella* abundance and DKD progression (Hu et al., 2025). However, whether *Rikenellaceae_RC9_gut_group*, *Parabacteroides*, and *Klebsiella* exert genuine protective effects in DKD and their mechanistic relationship with empagliflozin warrant further investigation.

Microbiota-targeted interventions have emerged as a key research focus in recent years, solidifying the gut microbiota’s functional role in DKD pathogenesis. For instance, polysaccharides from the Fufang Zhenzhu Tiaozhi formula alleviated renal pathological damage and reduced ACR in DKD mice by modulating the gut microbiota, elevating SCFA levels, and suppressing harmful bacterial overgrowth (Lan et al., 2023). Similarly, Lactobacillus rhamnosus GG supplementation reduced glycated hemoglobin, inflammatory factor levels, and urinary albumin excretion via gut microbiota reconstruction (Qumsani, 2025), while a probiotic cocktail (*Bifidobacterium bifidum*, *Lactobacillus acidophilus*, and *Streptococcus thermophilus*) effectively lowered ACR and delayed renal injury progression in DKD patients (Ghosh et al., 2024). Most notably, FMT from healthy to DKD mice directly reduced ACR and mitigated renal inflammation and fibrosis, establishing a clear causal link between microbiota remodeling and ACR regulation (Singh et al., 2020).

In addition to regulating gut microbiota, empagliflozin exerts renoprotective effects through multiple microbiota-independent mechanisms (O’Hara et al., 2024). These mechanisms include improving renal hemodynamics and reducing intraglomerular hypertension, attenuating glucose toxicity and oxidative stress, exerting direct anti-inflammatory effects, protecting mitochondrial function, and inhibiting renal fibrosis signaling pathways. Therefore, the sex-specific differences in renoprotection observed in our study may result from the combined effects of gut microbiota modulation and the direct renal protective effects of empagliflozin.

This study had several limitations. First, genetic background differences existed between C57 mice and db/db mice, as C57 mice were selected due to the unavailability of female db/m mice. Second, while we identified empagliflozin-associated gut microbiota and hypothesized their potential renoprotective effects, these effects were not definitively verified and will be the focus of our subsequent research. Third, the functional prediction of gut microbiota was only based on 16S rRNA sequencing data using the Tax4Fun tool, without validation by metabolomic or metagenomic experiments. Thus, the predicted KEGG pathway changes are only hypothetical and cannot fully reflect the actual functional status of the gut microbiota in DKD mice. In conclusion, we found that empagliflozin significantly reduced ACR levels and altered gut microbial richness and diversity in db/db mice, exerting sex-specific effects on gut microbiota composition. We additionally identified gut microbial genera significantly correlated with ACR. These findings suggest that empagliflozin may exert renoprotective effects by reshaping the gut microbial community structure. A deeper understanding of the interplay between SGLT2 inhibitors and the gut microbiota is critical for optimizing therapeutic strategies for DKD. In our subsequent research, we will focus on conducting *in vivo* experiments (e.g., FMT, SCFA supplementation, intestinal barrier disruption models) and *in vitro* cell experiments (e.g., renal tubular epithelial cells/podocytes co-cultured with microbial metabolites) to systematically elucidate the molecular mechanisms by which empagliflozin modulates the gut microbiota to exert renoprotective effects. Combined with multi-omics technologies including metabolomics and transcriptomics, we will further identify the key microbial metabolites and signaling pathways involved in this process.

## Data Availability

All raw sequencing data have been deposited in the NCBI Sequence Read Archive (SRA) under BioProject PRJNA1469662.

## Footnotes

1
https://magic.novogene.com/
