# Supplementary material for: Sex-specific modulation of gut microbiota by empagliflozin contributes to renoprotection in diabetic kidney disease mice
Source: Front Microbiol. 2026 Jun 5;17:1790953. doi: 10.3389/fmicb.2026.1790953 (PMC13278939; doi:10.3389/fmicb.2026.1790953)
Supplement: Supplementary file 1 [file Table_1.docx]

Supplementary Material

# Supplementary Table 1

Blood and urinary biochemical parameters in each mouse group.

|  | NC.F | NC.M | DM.F | DM.M | Empa.F | Empa.M |
| --- | --- | --- | --- | --- | --- | --- |
| TG | 0.499  (0.437, 0.598) | 0.577  (0.502, 0.633) | 1.161  (0.875, 2.177) | 1.423  (0.948, 2.102) | 1.249  (1.136, 1.399) | 1.182  (0.867, 1.407) |
| Chol | 2.290  (2.199, 3.001) | 2.721  (2.679, 2.772) | 4.155  (3.527, 4.898) | 4.092  (3.770, 4.717) | 3.741  (3.207, 3.851) | 3.566  (3.414, 4.042) |
| HDL | 0.587  (0.481, 0.665) | 1.138  (1.063, 1.198) | 1.055  (0.704, 1.184) | 1.458  (0.936, 1.856) | 0.670  (0.426, 1.010) | 0.604  (0.388, 1.792) |
| LDL | 0.577  (0.508, 0.851) | 0.588  (0.473, 0.642) | 0.905  (0.786, 1.059) | 0.795  (0.549, 1.018) | 1.676  (1.068, 2.454) | 1.225  (0.978, 1.964) |
| GSP | 2.786  (2.374, 3.671) | 2.322  (2.251, 2.380) | 4.911  (3.645, 5.765) | 3.820  (3.491, 4.643) | 2.417  (0.868, 3.898) | 3.982  (3.129, 4.301) |
| ACR | 33.7  (16.8, 47.5) | 33.5  (23.7, 71.6) | 107.0  (74.3, 115.0) | 216.0  (167.0, 247.0) | 47.5  (37.5, 55.1) | 117.0  (97.5, 124.0) |
| Urea | 19.69  (19.35, 22.03) | 25.64  (21.24, 28.24) | 18.75  (16.03, 24.14) | 27.03  (25.14, 30.44) | 24.90  (23.11, 27.16) | 24.81  (20.49, 29.46) |
| Cr | 37.51  (26.12, 49.96) | 44.21  (41.50, 49.56) | 29.34  (25.02, 32.72) | 32.42  (22.87, 35.49) | 24.96  (21.23, 27.20) | 31.08  (29.19, 37.15) |

Abbreviations: TG, triglyceride; Chol, cholesterol; HDL, high-density lipoprotein; LDL, low-density lipoprotein; GSP, glycated serum protein; ACR, albumin-to-creatinine ratio; Urea, urea nitrogen; Cr, creatinine.
